# A vanished gastric gastrointestinal stromal tumor

**DOI:** 10.1186/s40792-023-01674-z

**Published:** 2023-05-31

**Authors:** Sarah Honjo, Suguru Yamauchi, Yutaro Yoshimoto, Chen Jun, Hiroki Egawa, Akira Kubota, Kenki Tsuda, Yukinori Yube, Sanae Kaji, Hajime Orita, Tetsu Fukunaga

**Affiliations:** 1 Department of Esophageal and Gastroenterological Surgery, Juntendo University Hospital, 3-1-3 Hongo, Bunkyo-Ku, Tokyo, 113-8431 Japan; 2 Department of Surgery, Johns Hopkins University School of Medicine, Baltimore, MD 21287 USA

**Keywords:** Gastrointestinal stroma tumor (GIST), Vanished, Spontaneous regression, Laparoscopy and endoscopy cooperative surgery (LECS)

## Abstract

**Background:**

Local resection is the standard treatment for gastrointestinal stromal tumors (GISTs). Laparoscopic and endoscopic cooperative surgery (LECS) is a minimally invasive surgery used to resect GISTs. Herein, we report an extremely rare case of a gastric GIST that grossly vanished during LECS.

**Case presentation:**

A 50-year-old Japanese female was referred to our hospital after an abnormality was detected during an esophagogastroduodenoscopy (EGD) at her annual health checkup. Based on EGD, endoscopic ultrasound (EUS), and computer tomography (CT) findings, the patient was diagnosed with a 50-mm submucosal tumor (SMT) with intraluminal growth on the anterior wall of the lesser curvature of the upper body of the stomach. We routinely use LECS to treat the intraluminal growth type of GISTs. During the intraoperative endoscopy, the intraluminal submucosal tumor, which was detected preoperatively, had vanished. A red-white scar was observed in the regressed tumor region. LECS was performed by resecting at a distance away from the scar tissue and closing the gastric wall with intracavitary sutures. In the evaluation from the tumor section view of the original resected specimen, a 22 × 14 × 8 mm lobular neoplasm was observed that was predominantly located in the gastric submucosa to the muscularis propia. Pathological findings confirmed the diagnosis of GIST with intermediate risk indicated by the Fletcher classification. The patient continued postoperative adjuvant chemotherapy with imatinib and no recurrence was detected over 12 months after surgery.

**Conclusion:**

LECS was performed on the vanished gastric GIST, providing the best surgical treatment and leading to an accurate diagnosis and optimal postoperative care.

## Background

Gastrointestinal stromal tumors (GISTs) are the most common mesenchymal tumors of the gastrointestinal tract, yet they account for only 1–3% of all gastrointestinal tumors [1]. Local resection is the standard treatment for GISTs, and various laparoscopic techniques for resection have been developed. Laparoscopic and endoscopic cooperative surgery (LECS) is a minimally invasive surgery applied to resect GISTs [2]. LECS, an intraoperative endoscopic procedure, allows for tumor removal with minimal resection, leading to organ preservation and maintenance of postoperative quality of life. We report an extremely rare case of gastric GIST that grossly vanished during LECS.

## Case presentation

**Fig. 1:**
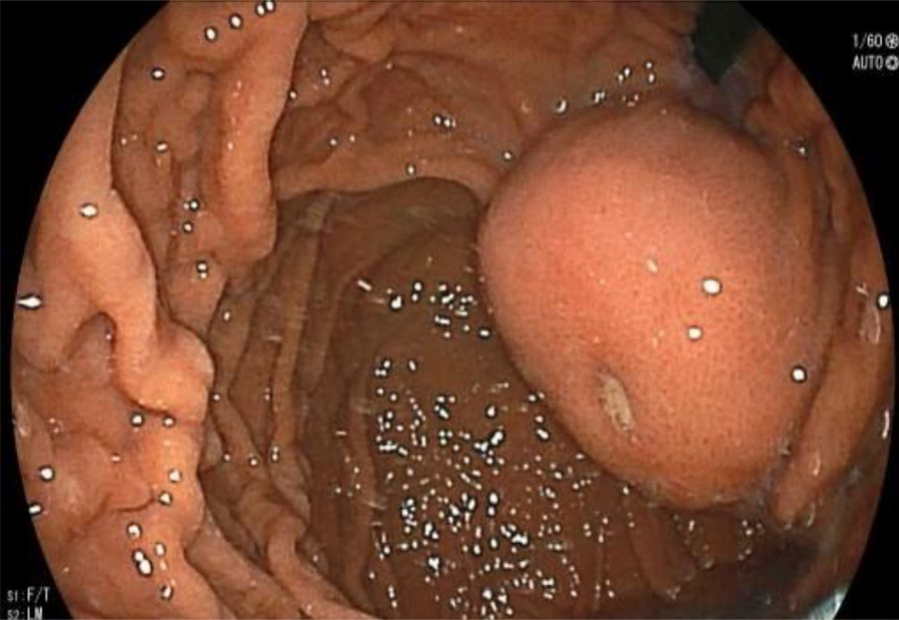
Esophagogastroduodenoscopy revealed a submucosal tumor of over 50 mm with central ulceration on the anterior wall of the lesser curvature of the upper body of the stomach

**Fig. 2:**
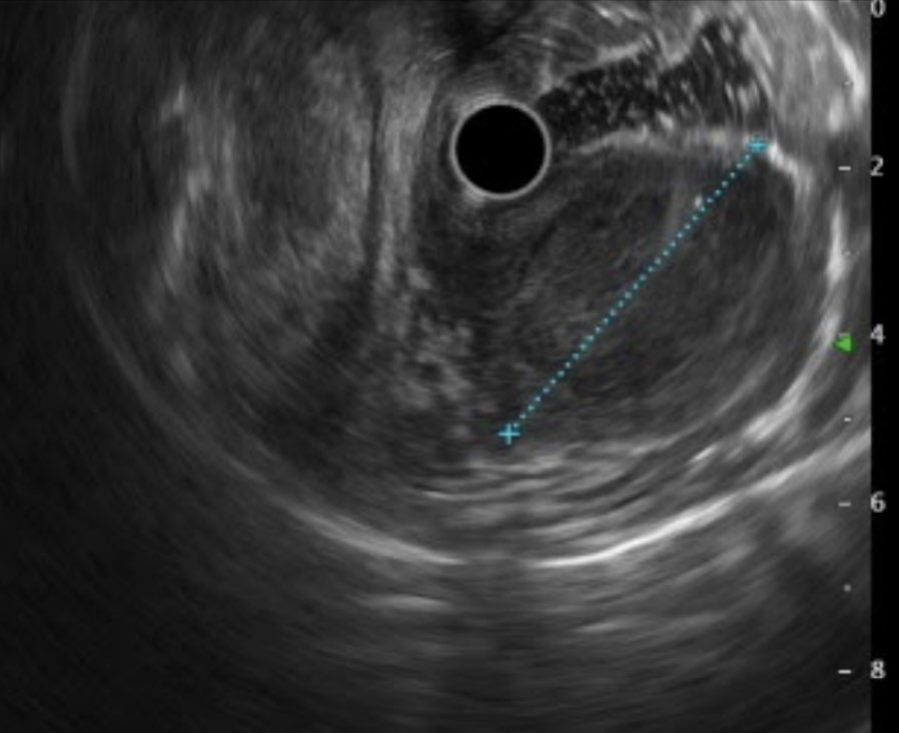
Endoscopic ultrasound revealed a 50-mm oval heteroechoic tumor arising from the muscularis propria (fourth layer) with a slight blood flow signal

**Fig. 3:**
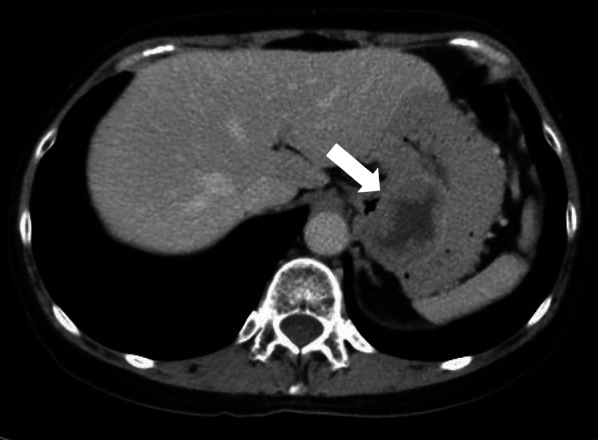
Computer tomography showed a 50 mm × 36 mm ovoid, well-marginated, intraluminal, heterogeneous mass at the upper body of the stomach, without invasion of adjacent organs or tissues (white arrow)

A 50-year-old Japanese female with a past medical history of chronic obstructive pulmonary disease and bronchial asthma was referred to our hospital following the detection of an abnormality on esophagogastroduodenoscopy (EGD) at her annual health checkup. EGD revealed a submucosal tumor of over 50 mm with central ulceration on the anterior wall of the lesser curvature of the upper body of the stomach (Fig. 1). Endoscopic ultrasound (EUS) revealed a 50-mm oval heteroechoic tumor arising from the muscularis propria (fourth layer) with a slight blood flow signal (Fig. 2). EUS-guided fine needle aspiration (EUS-FNA) was not performed because of the size of the submucosal tumor and the central ulceration. The malignant findings were an indication for surgery. Computer tomography (CT) showed a 50 mm × 36 mm ovoid, well-marginated, intraluminal, heterogeneous mass at the upper body of the stomach, without invasion of adjacent organs or tissues (Fig. 3). Thus, a provisional diagnosis of an intraluminal growth type of submucosal tumor was made.

**Fig. 4:**
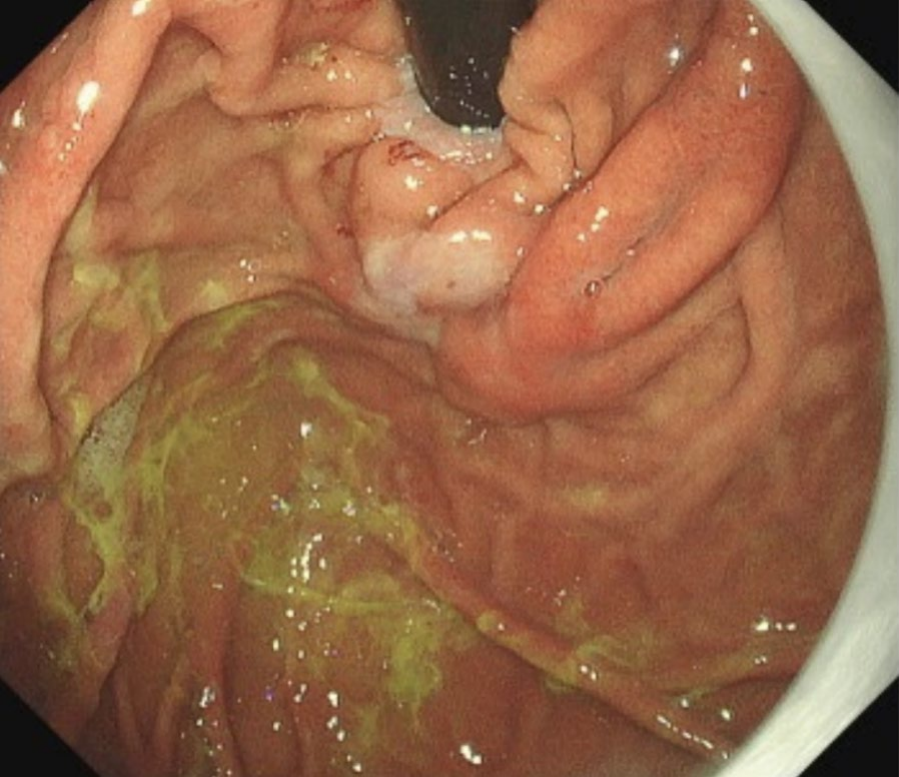
The previously detected intraluminal growth submucosal tumor was no longer visible. A red and white scar was observed in the regressed tumor region

**Fig. 5:**
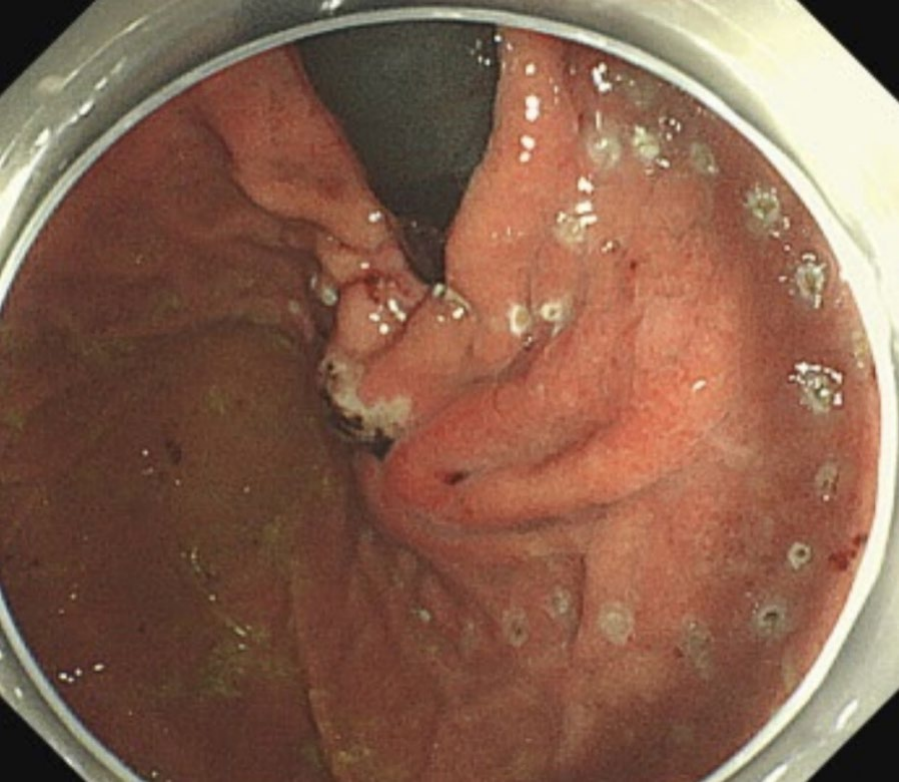
Laparoscopic and endoscopic cooperative surgery was performed by resecting at a distance away from scar tissue and closing the gastric wall with intracavitary sutures

**Fig. 6:**
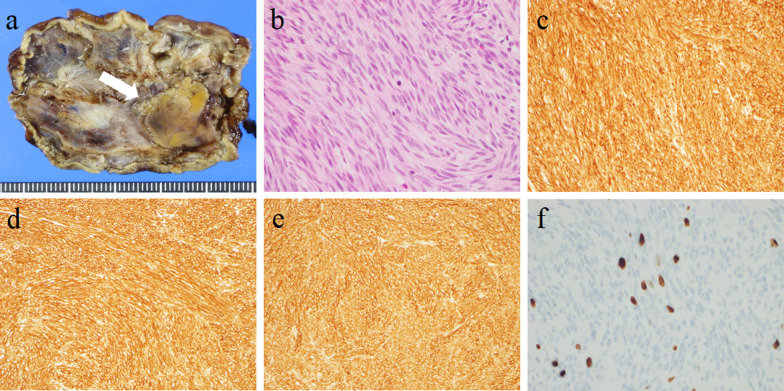
**a** A 22 × 14 × 8 mm lobular neoplasm was observed that was predominantly located in the gastric submucosa to the muscularis propia (white arrow). **b** Microscopic examination showed bundle-like proliferation of spindle-shaped cells in the tumor. **c** The tumor was positive for c-kit. **d** The tumor was positive for DOG-1. **e** The tumor was positive for CD34. **f** MIB-I index was 8%

LECS were performed. Under general anesthesia, 5 trocars were inserted, including a 12-mm umbilical trocar, a 12-mm trocar at the left subcostal, and 3 5-mm ports inserted to form a shallow trapezoidal shape. During the intraoperative endoscopy, the previously detected intraluminal growth submucosal tumor was no longer visible. A red and white scar was observed in the regressed tumor region (Fig. 4). Because of the possibility of a tumor remnant, the resection was continued. LECS was performed by resecting at a distance away from scar tissue and closing the gastric wall with intracavitary sutures (Fig. 5). To prevent intraoperative tumor dissemination, extreme care was taken to ensure that the relevant gastric mucosa was not exposed to the abdominal cavity. The operation time was 322 min and the blood loss was 10 ml. No postoperative complications occurred. The patient was discharged in stable condition on postoperative day 7. A figure of the tumor sectional view of the resected specimen was shown to clearly illustrate the presence of the tumor (Fig. 6a). A 22 × 14 × 8 mm lobular neoplasm was observed that was predominantly located in the gastric submucosa to the muscularis propia. Microscopic examination showed bundle-like proliferation of spindle-shaped cells in the tumor (Fig. 6b). The mitotic count was 8/50 high power fields. According to the immunohistochemical analysis, the tumor was positive for c-kit (Fig. 6c), DOG-1 (Fig. 6d), CD34 (Fig. 6e), with a MIB-I index of 8% (Fig. 6f). Thus, the histology results confirmed the diagnosis of GIST with a modified Fletcher classification indicating intermediate risk. The patient continued postoperative adjuvant chemotherapy with imatinib and no evidence of recurrence was observed after more than 12 months of postoperative follow up.

## Discussion

No previous reports described spontaneously vanishing GISTs, and this was an extremely rare case. The tumor, which grossly disappeared intraoperatively, was resected without excess or deficiency by LECS, leading to an accurate diagnosis and optimal postoperative treatment.

The terms spontaneous remission and spontaneous regression (SR) exist in the field of oncology [3]. SR of a malignant tumor is defined as the disappearance of part of the tumor or the entire tumor despite no treatment or inadequate curative treatment [4]. Several reports describe SR of neuroblastomas, renal cell carcinomas, malignant melanomas, and lymphomas, although the SR of a GIST has not been reported [5]. In this case we experienced, there has been no definitive conclusion as to whether the terms “spontaneous remission” or “regression” are appropriate to apply. The indisputable fact is that the tumor, which was over 50 mm in size at the time of initial diagnosis, appeared as a 20-mm tumor at the time of removal without treatment. The possible progression of the tumor if left untreated cannot be determined. However, the crucial point is that the tumor morphologically changed and was no longer visible to the naked eye. Because of this phenomenon, designing a treatment plan was difficult.

Endoscopy, EUS, and CT are commonly used to diagnose GISTs [6]. In our case, preoperative imaging with endoscopy, EUS, and CT revealed typical findings of gastric GIST with a large tumor size. Thus, EUS-FNA was not performed, and surgery was decided as the first-line treatment. According to the Japanese guidelines for GIST treatment, surgery is the first choice for submucosal tumors larger than 5 cm, and a definitive diagnosis by preoperative biopsy is not always necessary [7].

The objective of surgery for localized GISTs is to achieve R0 resection to the greatest extent possible. Lymph node dissection is not recommended, except when lymph node metastasis is clinically suspected. Therefore, wedge or segmental resection with preservation of organ function to maintain quality of life is recommended [8]. Previous studies demonstrated that laparoscopic resection is feasible and safe for gastric GISTs and is less invasive than traditional open surgery, and laparoscopic and open surgeries for GISTs have similar oncologic outcomes [9].

LECS is the standard surgical procedure for GIST treatment because it is minimally invasive and adequate resection can be achieved [2, 10, 11]. Laparoscopic surgeries for GISTs larger than 5 cm have better short-term outcomes in terms of operating time, blood loss, perioperative complications, and hospital stays, and comparable long-term outcomes in terms of disease-free survival and overall survival [12]. At our institution, LECS is the first-line treatment for the intraluminal growth type of GISTs, regardless of size.

The presumed causes of tumor morphology changes, as in the present case, include hemorrhage, infection, necrosis, penetration, and perforation. Several reports described such conditions in patients with GISTs, but all of these conditions caused acute abdominal symptoms requiring emergency treatment[13, 14]. Furthermore, none of these conditions were associated with changes in tumor morphology. In our case, the patient showed no clinical signs of abdominal pain, fever, hematemesis, hematemesis, hemorrhage, or progressive anemia during the one month between the time of endoscopy, EUS, CT, and surgery. The pathology also showed no secondary findings suggestive of hemorrhage, infection, necrosis, or perforation.

Surprisingly, no obvious mass could be observed during the intraoperative endoscopy. The surgical team had to make a very difficult decision as to whether to resect or to stop the surgery and follow up. The decision about what area to resect was also challenging. Considering the possibility of a tumor remnant, the decision was reached to resect the tumor scar. Although no previous reports describe the extent of resection, we performed full-thickness resection with a margin of 2–3 cm from the probable tumor scar by LECS. As a result, the histopathological diagnosis of GIST was confirmed, the resection margin distance was not excessive, and the best surgical treatment was provided. Even after attentive observation of the resected specimen, it was incapable of noting the presence of the tumor from the mucosal surface grossly, and a 22 mm mass was identified histologically in the gastric submucosa to the muscularis propia. A retrospective review suggested that the tumor could not have been identified by conventional endoscopy at the time the LECS was performed, although its presence could have been confirmed by EUS. Additionally, the EUS findings would have been very useful in deciding to resect and determining the extent of the resection. Performing EUS routinely during LECS is not practical due to the need for special equipment and scopes. Nonetheless, in situations similar to this case during LECS, EUS can be a valuable strategy.

## Conclusion

We experienced a very rare case of a vanished gastric GIST. LECS enabled the best surgical treatment, leading to an accurate diagnosis and optimal postoperative care.

## Data Availability

Data sharing is not applicable to this article as no datasets were generated or analyzed during the current study.

## Abbreviations

GIST: Gastrointestinal stromal tumor
LECS: Laparoscopic and endoscopic cooperative surgery
EGD: Esophagogastroduodenoscopy
EUS: Endoscopic ultrasound
EUS-FNA: EUS-guided fine needle aspiration
CT: Computer tomography
SMT: Submucosal tumor
SR: Spontaneous regression
